# Knowledge Representations Derived From Semantic Fluency Data

**DOI:** 10.3389/fpsyg.2022.815860

**Published:** 2022-03-11

**Authors:** Jeffrey C. Zemla

**Affiliations:** Department of Psychology, Syracuse University, Syracuse, NY, United States

**Keywords:** semantic fluency, semantic representation, semantic network, Alzheimer’s disease, semantic memory

## Abstract

The semantic fluency task is commonly used as a measure of one’s ability to retrieve semantic concepts. While performance is typically scored by counting the total number of responses, the ordering of responses can be used to estimate how individuals or groups organize semantic concepts within a category. I provide an overview of this methodology, using Alzheimer’s disease as a case study for how the approach can help advance theoretical questions about the nature of semantic representation. However, many open questions surrounding the validity and reliability of this approach remain unresolved.

## Introduction

Thinking about a concept (such as *table*) automatically and implicitly primes semantically related concepts (such as *chair* and *desk*). This occurs because the mind organizes concepts in an associative manner, called a semantic representation (Collins and Loftus, 1975; Shepard, 1980; Günther et al., 2019; Kumar, 2021). Though this representation is not directly observable, it can be estimated from psychological data. Accurately estimating representations has become an important computational problem in psychology, in part because it may help us understand the underlying nature of knowledge impairments. Why do individuals with Alzheimer’s disease have difficulty retrieving semantic knowledge? Why do individuals with schizophrenia have disorganized thought? While these impairments are complex and multifactorial, one contributing factor may be how the minds of those individuals organize knowledge differently.

Semantic representations have been estimated from a number of behavioral tasks, including the free association task (e.g., De Deyne et al., 2019) and large text corpora (e.g., Jones and Mewhort, 2007). Here, I focus on another task used to estimate representations that has a long history in psychology: the semantic fluency task (Bousfield and Sedgewick, 1944). In the fluency task, participants list as many exemplars from a category (such as *animals*, *foods*, or *furniture*) as they can recall in a fixed period of time (typically 1–3 min). The *animals* category is the most widely studied, in part because of its presence in large datasets, such as the National Institute on Aging’s Uniform Data Set (Weintraub et al., 2009), and in other widely used neuropsychological tests, such as the Modified Mini-mental State Exam (Teng and Chui, 1987) and Boston Diagnostic Aphasia Examination (Goodglass et al., 2001). Performance on the semantic fluency task is generally correlated across different categories (Acevedo et al., 2000; Castro et al., 2021), though some categories may serve as more sensitive indicators of cognitive decline than others, perhaps due to category-specific impairments (Gocer March and Pattison, 2006; Moreno-Martínez et al., 2017). There is substantial evidence that the semantic fluency task uses fundamentally different cognitive processes than the letter fluency task (e.g., Baldo et al., 2010; Birn et al., 2010), but little is known about whether the cognitive processes and brain regions associated with the semantic fluency task are affected by category.

Performance on the task is traditionally measured by counting the number of responses, but the order of individual responses is not random. Participants typically generate consecutive responses that are semantically similar (e.g., cat, lion, and tiger). This occurs in part because of automatic, associative retrieval processes (Hills et al., 2015). As one response is generated (e.g., *cat*), it primes semantically associated responses (e.g., *lion*). This pattern makes it possible to draw inferences about the relatedness of concepts encoded in semantic memory. For example, *cat* and *lion* are likely to be close together in one’s representation of animals, whereas *cat* and *whale* are likely to be further apart. With enough fluency data, computational methods can be used to estimate a semantic representation.

While the semantic fluency task also involves controlled retrieval processes (associated with executive functioning), semantic priming in the fluency task is commonly assumed to reflect aspects of the semantic representation and not executive processes. Evidence from fMRI studies shows that frontal lobe activation in healthy adults is associated with transitions between non-semantically related responses, but not during free generation of semantically related items (Hirshorn and Thompson-Schill, 2006). Similarly, frontal lobe lesions do not affect semantically related transitions while lesions in the temporal lobe (associated with semantic processing) do (Troyer et al., 1998). Behavior on the fluency task is also largely unaffected by a dual task that requires executive functioning (Moscovitch, 1994). Nonetheless, many populations that are impaired on the semantic fluency task (such as those with Alzheimer’s disease) also have executive functioning deficits, making it diffiult to ascribe impaired behavior solely to semantic representation—this potential confound is revisited later.

Here, I provide an overview of how semantic representations estimated from fluency data have been used to improve our understanding of knowledge impairments, using Alzheimer’s disease as a case study. However, a number of methodological issues surrounding the reliability and validity of this approach remain to be resolved.

## Models of Semantic Representation

Semantic memory can be represented in many different ways. Two widely used classes of semantic representation^1^ are semantic networks (Borge-Holthoefer and Arenas, 2010; Baronchelli et al., 2013) and distributional semantic models^2^ (Günther et al., 2019). While both classes encode associations between concepts within a category, their formalisms differ.

Semantic networks encode associations as a set of nodes and a set of edges. Each node represents a concept, and each edge (connecting two nodes) represents an association between those concepts. The similarity between two concepts can be approximated by the distance between them in the network (e.g., two nodes that are connected by an edge are more similar that two nodes that are only indirectly connected). For example, *snake* and *lizard* may be directly connected by an edge because they are both *reptiles*, whereas *snake* and *elephant* might be indirectly connected through a series of edges. There are many variations of semantic networks, including those with edge weights that indicate association strength, and directed edges to indicate asymmetric similarity.

**Figure 1:**
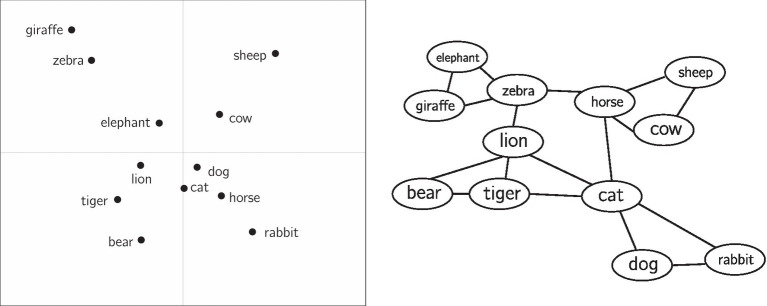
For illustrative purposes, a hypothetical distributional semantic model is shown (left) alongside a hypothetical semantic network (right). In the distributional semantic model, physical distance between items approximates similarity between concepts. The axes are in arbitrary units. In the semantic network, similarity is approximated by the paths (edges) connecting concepts. Here, the distributional semantic model is shown in two dimensions, though often these representations are in a higher-dimensional space. The left figure is re-created approximately from Chan et al. (1993), while the right figure has been mocked up for comparison.

In contrast to semantic networks, distributional semantic models represent each concept as a vector in a multidimensional space. The similarity between two concepts in a distributional semantic model can be approximated by the distance between the two vectors (e.g., cosine distance). Figure 1 depicts both a semantic network and distributional semantic model.

Semantic representations holistically encode the associations between a large set of concepts at once, because all concepts are situated within the same “space.” This allows for precise quantitative predictions about the association between two concepts. For example, similarity ratings (De Deyne et al., 2016), response times in word naming, lexical decision tasks, and relatedness judgments (Kenett et al., 2017; Mandera et al., 2017; Kumar et al., 2020; Levy et al., 2021), and word-level properties such as concreteness or valence (Vankrunkelsven et al., 2018) can be predicted from micro- or meso-level properties of the representation, such as the distance between two concepts in a representation or the number of nearby neighbors for a concept (Kenett and Faust, 2019b; Siew et al., 2019). It also allows for a macro-level description of the structure of associations. For example, semantic representations are often described as being clustered. In Figure 1 (right), there is a cluster of farm animals (*horse*, *cow*, *sheep*) that are close to each other, though visually distinct from other clusters. Community detection algorithms may be used to detect these clusters, while measures such as the network clustering coefficient can quantify the degree to which a network is clustered as a whole. Other measures such as average shortest-path length (for networks) or average inter-item distance (for distributional semantic models) quantify how dispersed concepts are within a representation.

Quantifying the macro-level structure of a representation is thought to be a useful way to understand how the mind encodes semantic knowledge. Measures derived from representations are used to describe differences in knowledge between groups (or individuals). For example, highly creative individuals may have representations that are more interconnected than less creative individuals (Kenett and Faust, 2019a), and this tightly interconnected structure may be what allows creative individuals to find associations between distantly related concepts (Rossman and Fink, 2010).

## The Semantic Fluency Task and Knowledge Impairments

The semantic fluency task is commonly used in neuropsychological batteries, including dementia screening (e.g., Weintraub et al., 2009). The most common way to score the task is to simply count the total number of responses listed by a participant. While this measure is effective at differentiating between groups with and without memory impairments, it provides little insight into the nature of the memory impairment and fails to identify and distinguish between different *types* of memory impairments. For example, individuals with either Alzheimer’s disease or Huntington’s disease reliably generate fewer responses in the semantic fluency task than age-matched controls (Randolph et al., 1993). Yet the locus of the impairment in these groups may differ; Rohrer et al. (1999) argue that semantic memory impairments in Alzheimer’s disease may primarily be a result of representational deficits, whereas impairments in Huntington’s disease may be caused by retrieval failures.

Other measures of scoring the semantic fluency task are used, though more commonly in research rather than in clinical settings. For example, measuring the number of perseverations (repetitions) generated in the task; measuring the average typicality of responses (e.g., *dog* is more typical than *aardvark* of the category animals); or measuring the average cluster size^3^ (i.e., the number of semantically related responses generated in sequence). These measures can be combined to form behavioral phenotypes that are better able to distinguish between knowledge types of memory impairments. For example, both Alzheimer’s disease and Parkinson’s disease are associated with fewer responses in the semantic fluency task, but only Alzheimer’s disease (and not Parkinson’s) is associated with generating smaller clusters (Troyer et al., 1998).

Still, this approach fails to offer a unified account of the data. What might cause individuals with Alzheimer’s disease to generate fewer responses *and* smaller clusters? The allure of semantic representations is that they offer a cognitive explanation for a complex pattern of results, one that characterizes differences in knowledge rather than differences in behavior. In addition, semantic representations offer a way to generating testable predictions.

## Estimating Semantic Representations From Fluency Data

There are a number of different algorithms used to estimate semantic representations from fluency data. Distributional semantic models have been estimated using multidimensional scaling (Shepard, 1980), with the distance between items in a fluency list (i.e., the number of intervening items) used as pairwise similarity ratings (Weakley and Schmitter-Edgecombe, 2014). Another approach is to use singular value decomposition on a binary participant by response matrix of responses (Sung et al., 2012), where each cell in the matrix indicates whether a particular word was listed by a participant. The output of these procedures is a vector space, in which each concept has its own coordinates.

Semantic networks are often estimated through a co-occurrence model. In the simplest version of this model, two concepts are connected by an edge if they appear adjacent to each other in a single fluency list (known as a naïve random walk; Jun et al., 2015). A variant of this model introduces edges between concepts that are not adjacent if they are within a window of each other. For example, in the fluency list *cat, lion, giraffe*, the concepts *cat* and *giraffe* would be directly connected by an edge given a window size of two (*giraffe* is within two responses of *cat*). To limit the number of false positives, a threshold is often used: two concepts must need to appear adjacent to each other in multiple fluency lists (the exact number or proportion of lists is a free parameter). The window size and threshold parameters are typically set by the researchers, though the threshold can also control for the probability of chance responding. For example, Goñi et al. (2011) use a binomial proportion confidence interval to connect two concepts by an edge only when the two responses occur adjacently (or within a window) more often than expected by chance alone, given the number of opportunities they have to be adjacent.

Less commonly, approaches other than co-occurrence models have been used to estimate semantic networks. For example, Zemla and Austerweil (2018) describe a generative process wherein fluency data is produced by the first hits in a random walk on a semantic network. Given this process, a Bayesian model is used to estimate the network most likely to have generated the fluency data. Correlation-based networks are also used (Kenett et al., 2013; Borodkin et al., 2016). In this approach, a binary participant by word matrix if formed, where each cell indicates whether a participant lists a word in their fluency list. Correlations between word vectors indicate whether two words are likely to occur in the same fluency list, regardless of ordinal position. A complete matrix of word-to-word correlations represents a fully connected, weighted network, and additional procedures are commonly use to binarize weights (e.g., Kenett et al., 2013). An overview of these and other methods used to estimate semantic networks is provided by Zemla and Austerweil (2018). Several open-source software packages are available that implement these procedures (Zemla et al., 2020; Christensen and Kenett, 2021).

### Use Case: Alzheimer’s Disease and Mild Cognitive Impairment

Many individuals with Alzheimer’s disease are impaired at semantic tasks, such as object naming (Bayles and Tomoeda, 1983; Reilly et al., 2011) and free association (Abeysinghe et al., 1990; Gollan et al., 2006; Zannino et al., 2021). However, the locus of this impairment has been debated. One possibility is that individuals with Alzheimer’s disease have an impaired semantic representation. Evidence in favor of this hypothesis comes from the observation that those with Alzheimer’s disease make semantic errors for the same concepts across varied experimental tasks. For example, items that are incorrectly named in a picture-naming task are also likely to be sorted incorrectly in a categorization task (Hodges et al., 1992). Others have argued that semantic impairments stem primarily from retrieval failures, not representational deficits. In support of this hypothesis, individuals with Alzheimer’s disease may show evidence of semantic priming equivalent to that of healthy controls, a sign that implicit semantic relations are still encoded even if explicit retrieval is difficult (Nebes et al., 1984; Nakamura et al., 2000). Subsequent work has found that in the early stages of Alzheimer’s disease, those with Alzheimer’s disease can exhibit hyperpriming—that is, semantic priming that is *greater* in those with Alzheimer’s compared to healthy controls (Giffard et al., 2001). Laisney et al. (2011) suggest that hyperpriming in those with Alzheimer’s disease may be caused by the loss of semantic features that are distinctive to a concept (a representational deficit). For example, if one forgets that a *zebra* has *stripes*, then *zebra* and *horse* seem more similar to each other—which may lead to larger priming effects.

The debate over whether semantic representations are impaired in individuals with Alzheimer’s disease is difficult to settle with clever experimental design alone. As a result, Chan et al. (1993) were among the first to advance this debate by developing a method for estimating representations from fluency data. The authors used both ADDTREE (Sattath and Tversky, 1977) and multidimensional scaling (Shepard, 1980) to estimate representations from animal fluency data generated by a group of Alzheimer’s participants and a group of cognitively healthy age-matched controls. They found that many semantically similar concepts (e.g., *dog* and *cat*) were further apart in the representation of the Alzheimer’s group, while less related concepts (e.g., *bear* and *cow*) were closer together. Further, they found that the representation the Alzheimer’s group did not cluster neatly into interpretable categories (e.g., *bear* was grouped with domestic animals). The authors conclude that individuals with Alzheimer’s disease encode “new, albeit abnormal associations” between concepts in semantic memory, leading to disorganized representations relative to the cognitively healthy group.

Since Chan et al. (1993), a number of research groups have continued to explore how Alzheimer’s disease affects one’s semantic representation. In each case, the overarching goal is the same: estimate semantic representations from fluency data, and compare representations between healthy and impaired groups. The methods vary markedly, but in most cases, the respective authors find support for the hypothesis that semantic representations differ in some way between healthy older adults and those with Alzheimer’s disease.

Lerner et al. (2009) estimated semantic networks for groups of people with Alzheimer’s disease, Mild Cognitive Impairment (a disorder that commonly, though not always, precedes dementia or Alzheimer’s disease), and healthy controls using a naïve random walk method (Jun et al., 2015). They found that the Alzheimer’s semantic network had a higher density of associations and was less “small-world-like” (Humphries and Gurney, 2008) than the cognitively healthy network. Bertola et al. (2014) also use the naïve random walk method to estimate semantic networks of Alzheimer’s, Mild Cognitive Impairment, and healthy participants. Unlike Lerner et al. (2009), they construct a unique network for each individual. Due to the nature of the naïve random walk procedure (estimating an edge between each adjacent fluency response), this resulted in semantic networks that were almost entirely linear. Still, many network properties differentiated between the three groups: those with Alzheimer’s disease had networks with fewer nodes and higher density. One limitation of the naïve random walk procedure for estimating semantic networks is that it assumes that all adjacent items in a fluency list reflect associations in one’s semantic representation, though these non-semantically related transitions (i.e., cluster switches; Troyer et al., 1997) may actually reflect executive functioning processes (Hirshorn and Thompson-Schill, 2006) that are independent of one’s semantic representation.

Nonetheless, Zemla and Austerweil (2019) corroborate these findings using a different method that incorporates an executive functioning component and does not assume all adjacent responses are a reflection of one’s semantic representation. They use a hierarchical Bayesian model to estimate individual networks for participants with Alzheimer’s disease and cognitively healthy controls and found that a number of network measures, including network density and small-world-ness, can distinguish between those with Alzheimer’s disease and healthy controls. In addition, they find that several network measures correlate with performance on the Mini-Mental State Exam (Folstein et al., 1975), a widely used neuropsychological test. Similar to Chan et al. (1993), they conclude that semantic representations of those with Alzheimer’s disease can be described as having “spurious associations between unrelated concepts.”

Chang et al. (2013) provide additional evidence that semantic representations differ between healthy controls and those with Alzheimer’s. They apply both multidimensional scaling and hierarchical clustering to animal fluency data from a group of individuals with Alzheimer’s disease and a group of cognitively healthy adults. They conclude that both algorithms produce interpretable clusters for healthy control participants, but less organized clusters for the Alzheimer’s group.

However, other work suggests that these representational differences are not as pronounced. Weakley and Schmitter-Edgecombe (2014) used multidimensional scaling on fluency data and initially found that a group of Alzheimer’s individuals had less organized representations. Like Chan et al. (1993), they found that the healthy control group clustered animals into well-defined categories while the Alzheimer’s group did not. Yet after applying additional spatial transformations to align group representations, they conclude that the two groups in fact have similar representations, despite performing differently on the fluency task. Nevado et al. (2021) estimate semantic networks for a group of individuals with Mild Cognitive Impairment using a co-occurrence procedure (Goñi et al., 2011). They find that network measures such as modularity and clustering coefficient are virtually indistinguishable between the Mild Cognitive Impairment and control groups. It may be that those with Mild Cognitive Impairment do not have impaired representations, or that such impairments are not easily detectable.

Despite considerable variation in their methods, these results collectively suggest that the semantic representations of those with Alzheimer’s disease may differ systematically from those of healthy controls. This contributes to the long-standing debate about whether representations are impaired in Alzheimer’s disease, and highlights the utility of estimating representations from fluency data. However, these publications include considerable variation in the methods: despite asking the same central theoretical question of whether semantic representations are impaired in Alzheimer’s disease, they use different representational forms (networks and distributional semantic models), different algorithms to estimate these representations, and different dependent measures to compare them. This makes it difficult to compare and integrate the contributions of each into a more cohesive theory about how representations degrade due to Alzheimer’s disease; a quantitative meta-analysis is not possible.

## Unresolved Methodological Issues

An increasing number of publications have used semantic fluency data to estimate knowledge representations. Still, a large number of issues in the field remain unresolved. Procedural norms for collecting data and estimating representations vary widely, and reliability and validity are not commonly assessed.

### Lack of Procedural Norms

Currently, there are few norms guiding the collection and use of fluency data in estimating representations. While some variance in procedure is expected, little is known about how these choices affect estimated representations.

One example of this is whether to estimate representations of individual participants, or whether to pool data across participants to generate group-level representations. The most common procedure is to estimate group-level representations, but this choice has been criticized on both statistical and theoretical grounds (Verheyen et al., 2016). Cognitively healthy adults may have little inter-individual variance in their representations, but this assumption seems less tenable for those with cognitive impairments, especially among those at different stages of impairment.

One reason that group-level representations are more common may be pragmatic: all of the methods to estimate representations require a large number of fluency lists, and it is difficult to collect a large number of semantic fluency lists (from the same category) for a single individual. Zemla and Austerweil (2018) use a repeated fluency task in which each individual performed the semantic fluency task multiple times in a single session. However, there are practice effects within a session that lead to systematic differences across multiple trials of the task (Zemla and Austerweil, 2017). An alternative is to have an individual repeat the fluency task but space the trials far apart (i.e., a longitudinal study). Apart from being logistically difficult, this approach implicitly assumes that semantic representations are stable across time. This assumption is unlikely to be true for many patient populations, including those with neurodegenerative diseases like Alzheimer’s disease. Even cognitively healthy subjects may re-organize their semantic knowledge with experience, or flexibly re-organize their representation across time and in different contexts.

Another procedural question is deciding on how much data to use in estimating representations: how many lists should be collected, and how much time should participants have to generate responses for each fluency list? One might expect that a representation will converge with increasing amounts of data, analogous to increasing the sample size of an experiment, but this is not always true: some methods for estimating representations do not converge with increasing amounts of data, even when the data are generated from a known systematic process (Zemla and Austerweil, 2018). Increasing the time allotted for the fluency task also poses potential issues. Providing more time may increase the number of responses (especially low-typicality exemplars). These low-typicality responses increase the number of concepts in the representation, but can have large impact on macro-level structure of the representation. As a result, it is virtually impossible to compare structural properties (such as clustering or average shortest-path length, in a semantic network) across publications.

As the amount of data used can have a large impact on the estimated representations, most studies correct for this, but in idiosyncratic ways. For example, Nevado et al. (2021) truncate the length of fluency lists across participants, so that each list is the same length (but perhaps containing different responses). Chan et al. (1993) select only a limited set of twelve items that are common between groups to estimate representations, so that each representation contains the same concepts. These approaches both discard data that is potentially informative. Other approaches use a null model for comparison, rather than discard data, but the null model is not consistent across publications. Zemla and Austerweil (2019) compare semantic networks of individuals to networks estimated from permuted fluency data (the null model that each item in a semantic fluency list is independent of the last). Kenett et al. (2014) compare estimated representations to a random network generated from an Erdős-Rényi process (the null model that representations have no systemic structure). Other approaches include permuting the group or diagnostic label of individuals (Nevado et al., 2021), or using additional spatial transformation (Weakley and Schmitter-Edgecombe, 2014) to a put representations in a common coordinate space.

Little is known about how these procedural decisions affect results, and currently, there is no consensus as to which procedures should be used. This makes it difficult not only to evaluate the contributions of a research study, but to compare results across studies. Even if the procedural norms for every given study are sound, it remains difficult to aggregate these findings into a cohesive theoretical framework. This *ad hoc* approach contrasts with some modeling efforts in psychology, such as the sequential sampling model paradigm, that have successfully unified a wide array of methods in a single framework (Bogacz et al., 2006; Ratcliff et al., 2016).

### Validity

Estimating semantic representations is useful to the extent that those representations are valid—that is, those representations should accurately reflect the associations between concepts that are encoded in the mind.

Claims about how representations differ between groups suffer from poor construct validity if there is no attempt to verify whether those estimated representations are an accurately reflection of one’s true semantic representation. One method for assessing construct validity is to use semantic representations to make predictions about behavior on a subsequent task. For example, a semantic representation can be used to make predictions about pairwise similarity judgments (Zemla and Austerweil, 2018), response times for semantic relatedness judgments (Kumar et al., 2020), or triadic comparison judgments (i.e., the odd-one-out task; De Deyne et al., 2016). Yet it remains uncommon to predict behavior in semantic tasks and instead rely only on face validity (the extent to which the methods seem reasonable).

Regardless of their predictive power, representations estimated from fluency data may not necessarily reflect one’s true semantic representation. The semantic fluency task also requires retrieval processes, and the extent to which the data reflect aspects of the representation or the retrieval process are still debated (Abbott et al., 2015; Jones et al., 2015). As a result, estimated representations may actually reflect an amalgam of representation and retrieval processes (i.e., a functional representation). This may not be a concern if one’s objective is to make predictions about behavior or classify participants into groups, but is relevant if one is trying to disentangle the contributions of representation and process (as is the case with Alzheimer’s disease). While disentangling representation and process poses theoretical challenges (Anderson, 1978), it is an active topic in cognitive network science (Castro and Siew, 2020; Hills and Kenett, 2021).

Semantic representations should also have high internal validity. If adjacent responses in fluency data are semantically related *because* people are searching through their semantic representations, then those representations should be able to predict the original data that was used to fit the model. For example, semantic representations can be used to make predictions about which transitions are likely to occur in fluency data (e.g., how often should *zebra* appear after *horse*), and these predictions can be compared to the observed data. Other fields of psychology routinely use estimated models to predict the data used to estimate that model (i.e., check model fit), such as posterior predictive checks in Bayesian modeling (Gelman et al., 1996).

In practice, it is rare to assess either construct or internal validity when estimating semantic representations from fluency data. Some estimation methods have been validated in prior papers (i.e., when they are first described), but representations are not typically validated in subsequent studies when those methods are applied. This step is crucial because even if a model produces a valid representation for one dataset it may not produce a valid representation for another. Validity may depend on data quality, sample size, and the population in question (e.g., healthy versus cognitively impaired participants).

One reason why validity checks are infrequent may be that semantic representations are not generative models in and of themselves (though sometimes a generative model can be used to estimate a representation; see Zemla and Austerweil, 2018). As such, there is no consensus for how psychological data should be simulated from a representation. Nonetheless, it is straightforward to hypothesize such a process; for example, by using the Luce choice rule to predict transition probabilities in fluency data from pairwise similarity distance in a semantic representation. Another challenge with performing a validity check is that the output space of fluency data is virtually constrained: a participant’s response could be anything at all, even a non-category response (known as an intrusion), or the participant may not make a response (i.e., terminate search). Even if a generative model is defined to make a precise prediction about how often *zebra* should follow *horse*, the actual data may have very few observations of *horse* with which to compare model predictions.

### Reliability

Another challenge is to ensure the reliability of these representations. If a group of participants complete the semantic fluency task at two time points, how stable is that representation, both in absolute terms (e.g., is the distance between *zebra* and *horse* the same in both representations?) and in broad, structural terms (e.g., do both representations have a similar clustering coefficient?) A common method for assessing reliability in other domains is to use cross-validation. For example, one might use half of the data to estimate the parameters of a model and compare them to parameters estimated from the other half of the data. A similar procedure could be performed to estimate the reliability of semantic representations. Such metrics could bolster credence of a study’s results, and could also provide a principled measure for choosing among the many methods for estimating semantic representations.

An alternative to cross-validation is to use test–retest reliability. This is performed by estimating multiple representations from the same sample of individuals at different timepoints. White et al. (2014) have found that test–retest reliability from representations estimated using multidimensional scaling is low, and question the utility of these representations.

Reliability metrics are not commonly used when estimating representations. One exception is the use of Kruskal’s stress statistic in multidimensional scaling (Chan et al., 1993; Weakley and Schmitter-Edgecombe, 2014). However, this statistic is a goodness-of-fit measure akin to R^2^; it measures how closely the scaling solution matches the original data and does not use an independent sample of data as in cross-validation or test–retest procedures. In order to make progress in the field and have trustworthy models, researchers need to agree on a standard for assessing validity and reliability that can be used as a benchmark for future studies.

## General Discussion

The semantic fluency task is frequently used as part of a neuropsychological test for cognitive impairments, but the task is traditionally scored by simply counting number of responses. Nonetheless, there is clear evidence that the order of individual responses can help us understand how semantic representations are organized.

Accordingly, there have been many efforts to estimate semantic representations from fluency data for both cognitively healthy individuals and special populations. One population that has received a lot of attention is those with Alzheimer’s disease. Despite considerable variability in the methods used, many studies have arrived at the same conclusion: individuals with Alzheimer’s disease do indeed have disorganized representations when compared with cognitively healthy controls. These studies have helped provide insight into a long-standing debate about whether Alzheimer’s disease impairs one’s semantic representation, or whether it merely impairs retrieval of concepts on an intact representation.

These studies provide evidence that estimating representations from fluency data is a useful tool to understanding how the mind organizes semantic concepts. Still, many challenges remain. In order to safeguard the theoretical integrity of these findings, researchers will need to continue to improve the methods used with special attention given to reliability and validity of their models. There is no consensus in the field as to which representations are most appropriate to estimate (e.g., a semantic network or distributional semantic model) or which methods should be used for estimating these representations (e.g., multidimensional scaling or singular value decomposition). While most of these methods used have undergone some form of initial validity testing, they are rarely tested for validity or reliability in the specific context that they are used. This is distinct from other modeling domains, where parameter reliability and validity checks are routinely performed and reported within the same study. As the field matures, it is necessary for future studies to follow suit: some form of reliability and internal validity measures should be reported for every study. For example, one might test the reliability of an estimated representation using split-half cross-validation and test its validity by estimating the likelihood of generating the fluency data from that representation. When methods are used that do not allow calculations of reliability or validity, results should be viewed with skepticism. The inclusion of reliability and validity tests will aid researchers in choosing the appropriate procedures, as those studies with the highest validity and reliability will be viewed as the most robust and used in subsequent work.

## Author Contributions

The author confirms being the sole contributor of this work and has approved it for publication.

## Conflict of Interest

The author declares that the research was conducted in the absence of any commercial or financial relationships that could be construed as a potential conflict of interest.

## Publisher’s Note

All claims expressed in this article are solely those of the authors and do not necessarily represent those of their affiliated organizations, or those of the publisher, the editors and the reviewers. Any product that may be evaluated in this article, or claim that may be made by its manufacturer, is not guaranteed or endorsed by the publisher.

## Footnotes

^1^Other classes of semantic representation exist, such as feature-based models (McRae et al., 1997) or connectionist models (Rogers and McClelland, 2004). However they are not commonly estimated from semantic fluency data and are not discussed further.

^2^Distributional semantic models are sometimes referred to as word embeddings or semantic spaces, though the connotations of these terms may differ. Here, I refer to all of these collectively as distributional semantic models.

^3^For historical reasons, the term *cluster* has multiple meanings. Here, the term *cluster size* refers to a property of the fluency data (e.g., the number of farm animals listed sequentially). This is distinct from a *clustered representation* (p. 2), which refers to a property of the representation itself, not the data. While the latter may be a cause of the former, the two meanings are not synonymous.
